# Complete Genome Sequence of *Kinneretia* sp. Strain DAIF2, Isolated from a Freshwater Pond

**DOI:** 10.1128/MRA.00003-21

**Published:** 2021-02-25

**Authors:** Jacqueline Hollensteiner, Ines Friedrich, Lucas Hollstein, Jan-Philipp Lamping, Kalina Wolf, Heiko Liesegang, Anja Poehlein, Robert Hertel, Rolf Daniel

**Affiliations:** a Genomic and Applied Microbiology and Göttingen Genomics Laboratory, Institute of Microbiology and Genetics, Georg-August University of Göttingen, Göttingen, Germany; b FG Synthetic Microbiology, Institute of Biotechnology, BTU Cottbus-Senftenberg, Senftenberg, Germany; University of Delaware

## Abstract

*Kinneretia* sp. strain DAIF2 was isolated from a eutrophic freshwater pond. The genome consists of a single chromosome (6,010,585 bp) with a GC content of 69.3%. The whole-genome-based phylogeny of DAIF2 revealed a closest relation to the genus *Kinneretia*.

## ANNOUNCEMENT

The Gram-negative *Kinneretia* sp. strain DAIF2 was isolated from a eutrophic pond in Göttingen, Germany. The sample (51°33′29″N, 9°56′41″E) was collected on 24 September 2018. The strain was enriched and isolated as described previously (1). DAIF2 was chosen for sequencing, since it was most similar at the 16S rRNA gene level to the genus *Kinneretia*, which was until now only represented by the type strain, Kinneretia asaccharophila DSM 25082 (2). For DNA isolation, DAIF2 was cultivated in PCa medium (peptone medium supplemented with 0.015% CaC1_2_ [3]) at 25°C. DNA was extracted using the MasterPure complete DNA and RNA purification kit (Epicentre, Madison, WI, USA) as described previously (1). Illumina sequencing libraries were constructed using the Nextera XT DNA sample preparation kit (Illumina, San Diego, CA, USA) and sequenced using a MiSeq instrument and reagent kit v3 (600 cycles), as recommended by the manufacturer (Illumina). For Nanopore sequencing, a separate batch of 1.5 μg high-molecular-weight DNA was used for library preparation by employing the ligation sequencing kit 1D (SQK-LSK109) and the native barcode expansion kit (EXP-NBD114; barcode 19) as described by the manufacturer (Oxford Nanopore Technologies, Oxford, UK). The MinION device Mk1B, the SpotON flow cell R9.4.1, and MinKNOW software v19.06.8 were used for sequencing (72 h) as recommended by the manufacturer (Oxford Nanopore Technologies). For demultiplexing and base calling, Guppy v3.0.7 (Oxford Nanopore Technologies) was applied. Default parameters were used for all software unless otherwise specified. Sequencing resulted in 3,208,102 300-bp Illumina reads and 5,612,523 Nanopore reads with a mean length of 1,631 bp. The Illumina reads were quality filtered using Trimmomatic v0.36 (4), and paired reads were joined with FLASH (5). The Nanopore reads were adapter and quality trimmed with a length cutoff of 10 kb using fastp v0.20.0 (6), resulting in 75,898 Nanopore reads with an *N*_50_ value of 31,759 bp. Together with the Illumina reads, a *de novo* hybrid assembly was performed using Unicycler v0.4.8 (7) in normal mode. The assembly revealed a single circular chromosome (6,010,585 bp) with a GC content of 69.28%. Coverages calculated with Qualimap v2.2.1 (8) using Bowtie 2 v2.3.5 (9) and minimap2 v2.17-r941 (10) were 127-fold (Illumina) and 204-fold (Nanopore). The Prokaryotic Genome Annotation Pipeline (PGAP) v4.11 (11) was used for automatic DAIF2 genome annotation. Annotation revealed 5,538 putative genes, 5,398 of which were protein coding. Moreover, 64 tRNA genes, 15 rRNA genes, 1 transfer-messenger (tmRNA) gene, and 3 noncoding RNA (ncRNA) genes were identified.

**FIG 1:**
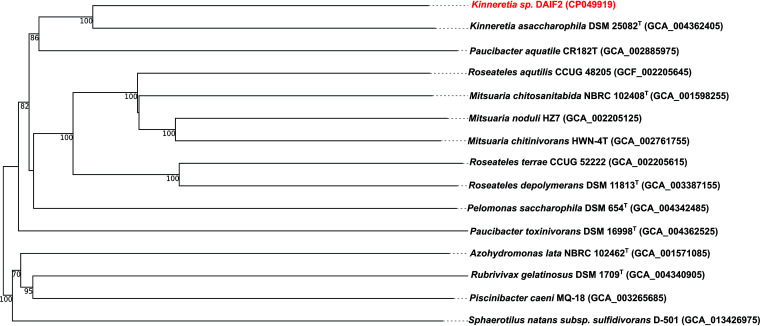
Phylogenetic classification of *Kinneretia* sp. strain DAIF2. The 14 closest related type strain genomes were used for phylogenetic analysis as described by TYGS (12). The tree was inferred with FastME 2.1.6.1 (13) using Genome BLAST Distance Phylogeny (GBDP) distances calculated from genome sequences. The branch lengths are scaled in terms of GBDP distance formula d5. The numbers above the branches are GBDP pseudobootstrap support values of >60% from 100 replications, with an average branch support of 85.8%. The tree was midpoint rooted (14).

Whole-genome-based phylogeny of the DAIF2 genome was performed with the Type (Strain) Genome Server (TYGS [12], accessed 12 November 2020). In general, close relationships of DAIF2 to the genera *Kinneretia*, *Paucibacter*, *Mitsuaria*, and *Roseateles*, which belong to the family *Comamonadaceae*, were detected (Fig. 1). The closest relative was the type strain Kinneretia asaccharophila DSM 25082 (GenBank accession number NZ_SNXE00000000.1) of the genus *Kinneretia*, which was announced in 2010 as a new genus in the *Rubrivivax* branch (2), with a calculated digital DNA-DNA hybridization (dDDH) of 34.5%. This result indicates that strain DAIF2 may be a new species (Fig. 1).

### Data availability.

This complete genome sequence is available at DDBJ/ENA/GenBank under the accession number CP049919.1. The raw reads were deposited in the NCBI sequence read archive (SRA) under the accession numbers SRX8059303 (Illumina) and SRX8059304 (Nanopore).
